# Multifaceted Rho GTPase Signaling at the Endomembranes

**DOI:** 10.3389/fcell.2019.00127

**Published:** 2019-07-16

**Authors:** Santosh Phuyal, Hesso Farhan

**Affiliations:** Department of Molecular Medicine, Institute of Basic Medical Sciences, University of Oslo, Oslo, Norway

**Keywords:** Rac1, Cdc42, RhoA, RhoB, RhoD, Golgi, endocytosis, membrane trafficking

## Abstract

The Rho family of small GTPases orchestrates fundamental biological processes such as cell cycle progression, cell migration, and actin cytoskeleton dynamics, and their aberrant signaling is linked to numerous human diseases and disorders. Traditionally, active Rho GTPase proteins were proposed to reside and function predominantly at the plasma membrane. While this view still holds true, it is emerging that active pool of multiple Rho GTPases are in part localized to endomembranes such as endosomes and the Golgi. In this review, we will focus on the intracellular pools and discuss how their local activation contributes to the shaping of various cellular processes. Our main focus will be on Rho signaling from the endosomes, Golgi, mitochondria and nucleus and how they regulate multiple cellular events such as receptor trafficking, cell proliferation and differentiation, cell migration and polarity.

## The Rho GTPase Family

The founding member of the Rho GTPase, termed Rho for Ras homolog, was identified in 1985 (Madaule and Axel, 1985). Shortly thereafter, two back-to-back papers elegantly demonstrated the functional importance of Rho and Rac in actin cytoskeleton assembly (Ridley and Hall, 1992; Ridley et al., 1992) driving the expansion of Rho GTPase biology. Since then, the Rho family of small GTPases has grown to include 20 separate proteins divided into seven subfamilies: Rho, Rac, Cdc42, Rnd, RhoD, RhoBTB, and RhoH (Hodge and Ridley, 2016). The majority of Rho family members undergo conformational switching between GTP-bound active and GDP-bound inactive states. This GTP/GDP cycling is tightly regulated by guanine nucleotide exchange factors (GEFs) and GTPase-activating proteins (GAPs). GEFs function as Rho GTPases activator by catalyzing the exchange of GDP for GTP, whereas GAPs facilitate hydrolysis of GTP leading to their inactivation. An additional layer of complexity is provided by guanine-nucleotide dissociation inhibitors (GDIs) that bind to the inactive pool of Rho GTPases and sequester them in cytosol (Garcia-Mata et al., 2011). Additional factors contributing to the complexity of Rho GTPase signaling is the crosstalk between its family members, distinct subcellular distribution of their GEFs and GAPs, and the post-transcriptional modifications such as phosphorylation, ubiquitination, and palmitoylation that regulate stability and spatial distribution of Rho GTPases.

The switch I and switch II domains of Rho GTPases undergo conformational change upon GTP-binding. Once active, Rho GTPases associate with membranes and selectively interact with downstream effectors and other scaffolding proteins to mediate a myriad of biological processes including reorganization of the actin cytoskeleton, regulation of membrane trafficking, cell motility and polarity. A large body of literature deals with signaling initiated by Rho GTPases and their effectors at or around the plasma membrane although intracellular pool of Rho GTPases and their regulators are increasingly apparent. It is becoming clear that coordination of functionally active Rho GTPases, their GEFs and GAPs, as well as their effectors extend beyond the plasma membrane to multiple intracellular organelles. Understanding how cells decode these spatio-temporal signals to generate biologic outputs is a major area of investigation.

Here, we review emerging knowledge on biological processes and signaling events mediated by intracellular pools of Rho GTPases, with special emphasis on signaling patterns that are triggered or initiated by endomembrane localized Rho GTPases. In particular, we summarize recent studies that provide direct evidence for endosomal, Golgi, mitochondrial and nuclear pools of Rho GTPase signaling.

## Rho GTPase Signaling From Endosomes

**FIGURE 1:**
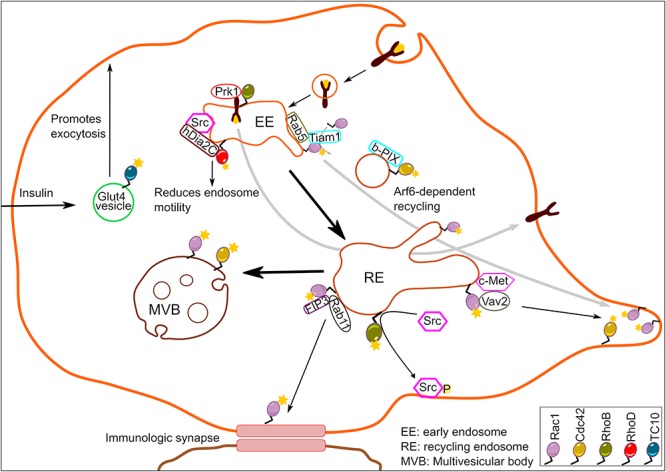
Schematic illustration depicting Rho GTPase signaling from endosomes. A number of Rho family members residing at endosomes generate localized signaling output to regulate a wide array of biological functions.

The endosomal system consists of pleomorphic membranous carriers that processes and transports a range of cargoes including active signaling receptors. The ability of endocytic organelles in generating compartmentalized signaling patterns by directional shuttling and/or retaining of signaling molecules into specific locations within the cell is well documented and has been extensively reviewed elsewhere (Palfy et al., 2012). In this review, we will summarize Rho GTPase signaling originating directly from the endosomal system and elaborate on how Rho family members utilize endosomes for signal propagation along the endocytic pathway (Figure 1).

From the Rho GTPase family, RhoB was among the first ones to be predominantly detected in the endocytic compartments (Adamson et al., 1992; Gampel and Mellor, 2002). Today, the list of endosome localized Rho GTPases has grown to include RhoD, Rac1, Cdc42, TCL, and TC10, although the underlying mechanism of their recruitment to endosomes remains to be clarified in the majority of cases. In the case of RhoB, the type of prenylation was suggested to be the major determinant of its precise localization. The geranylgeranylated form of RhoB was localized to late endosomes, while the farnesylated form was detected predominantly at the plasma membrane (Wherlock et al., 2004). Whether the kind of lipid modification is a deciding factor for all other endosome localized Rho GTPases remains to be addressed. Furthermore, the identity, localization and regulation of the prenylation factors responsible for differential RhoB prenylation is also poorly characterized. Additionally, it is also unclear how GEFs/GAPs for Rho GTPases are targeted to the endosomes. Nevertheless, the recruitment of Rho GTPases to endosomes generates spatially restricted signals that, as we will discuss in the following sections, has consequences for numerous cellular processes.

It can be expected that endosome-specific Rho GTPases represent a functionally distinct subset from those existing at the plasma membrane. Indeed, the farnesylated pool (plasma membrane localized) of RhoB appeared functionally distinct from the geranylgeranylated (endosome localized) pool in that treatment of cells with farnesyl-transferase inhibitors, which abolishes the farnesylated pool of RhoB, resulted in an increased recycling of endocytosed epidermal growth factor (EGF) receptor (Wherlock et al., 2004). In agreement with this, EGF-triggered signaling was prolonged in cells treated with farnesyl-transferase inhibitors. Endosomal RhoB was also shown to recruit the Ser/Thr kinase PRK1 to this compartment, resulting in its activation. Active PRK1 on endosomes then regulated the trafficking of EGFR in a way leading to prolonged signaling and preventing its degradation (Mellor et al., 1998; Gampel et al., 1999). Interestingly, PRK1 has been reported to phosphorylate the intermediate filament proteins vimentin and neurofilament as well as interact with α-actinin (Mukai, 2003). Whether RhoB-PRK1 regulated kinetics of EGF signaling shows actin-dependency remains to be explored in greater details. The fact that RhoB promotes endosome recycling appears to be true in several cell types. While RhoB controls EGFR recycling in epithelial cells, it also controls recycling of the integrin LFA-1 in T-lymphocytes, which regulates their migration (Samuelsson et al., 2017). These studies imply that RhoB targeted to endosomal compartment is an autonomous signaling entity. Supporting this view, cytoplasmic endosomes harboring RhoB were found to pick up inactive Src kinase and stimulate its activity *en route* to the plasma membrane (Sandilands et al., 2004). This type of translocation and activation of Src required the presence of RhoB containing endosomes since inactive pool of Src kinase accumulated around the perinuclear region in RhoB^–/–^ cells growing on fibronectin (Sandilands et al., 2004). The exact molecular mechanism underlying RhoB mediated endosomal motility, however, remains elusive. Proteins central to actin dynamics such as Scar1, Dia1, and mDia2 are also associated with RhoB-positive endosomes (Sandilands et al., 2004; Fernandez-Borja et al., 2005; Wallar et al., 2007) suggesting that RhoB-positive cytoplasmic entities could regulate endosomal trafficking in a manner dependent on rearrangement of actin cytoskeleton. It is currently unclear which GEFs activate RhoB on endosomes and which GAPs control this activity. In addition, it is unknown whether endocytic Rab GTPases contribute to the regulation of the endosomal RhoB pool. A possible candidate is the Rho-specific GAP, DLC3 which has been localized to Rab8-positive membrane tubules, reminiscent of the endocytic recycling compartment (Braun et al., 2015). Depletion of DLC3 impairs transferrin receptor endocytosis and this effect was neutralized by simultaneous depletion of RhoA and RhoB.

Several Rho family members regulate some aspects of actin cytoskeleton dynamics, and accordingly, multiple studies have demonstrated the importance of endosomal pool of Rho GTPases for actin-based endocytic vesicle movement. For example, a role for RhoD in controlling the endocytic vesicle movement has been documented. It was shown that cells possessing active RhoD display reduced velocity of early endosome movement, thereby slowing down the membrane trafficking events (Murphy et al., 1996). This effect of RhoD likely involves actin-based mechanisms since RhoD-mediated recruitment of its effector hDia2C to early endosomes aligns them along actin filaments (Gasman et al., 2003). RhoD has also been reported to bind to the Rab5 effector Rabankyrin-5 on early endosomes (Nehru et al., 2013). Thereby, Rab5 and RhoD may cooperate to regulate internalization of EGFR. It remains to be tested if RhoB and RhoD might control bidirectional endosomal movement by directly regulating each other’s activity. Such a crosstalk between Rho GTPases has already been demonstrated for RhoB and Rac1 (Marcos-Ramiro et al., 2016). In cytokine pre-treated vascular and microvascular endothelial cells, endosomal RhoB retains Rac1 in endosomes and negatively affects its activity to prevent endothelial barrier reformation (Marcos-Ramiro et al., 2016).

While RhoD and Rab5 cooperate to regulate endocytic trafficking, Rac1 required Rab5 for its endosomal recruitment (Palamidessi et al., 2008). Rac1 was detected by several groups on endosomes in different eukaryotic organisms (Strehle et al., 2006; Palamidessi et al., 2008; Menard et al., 2014). It was shown that formation of Rab5-positive early endosomes was a pre-requisite for endosomal recruitment of Rac1 and its GEF Tiam1 leading to Rac1 activation. This active, endosomal pool of Rac1 was then delivered to specific plasma membrane domains turning them into regions of localized actin cytoskeleton remodeling. The process of endocytic Rac1 delivery required another small GTPase, Arf6 (Figure 1). The Rab5-Rac1-Arf6 signaling circuit was crucial not only for localized actin dynamics and the morphology of cancer cells, but also for directed cell migration (Palamidessi et al., 2008). An analogous mechanism was found to account for concentration of Cdc42 at the leading edge of astrocytes (Osmani et al., 2010). In migrating astrocytes, Cdc42 and its GEF β-PIX were shown to co-localize on endosome-like structures. The localization of Cdc42 on endosome-like vesicles required Rab5, and the directed delivery of Cdc42 to the leading edge depended on Arf6, as was observed for Rac1. Hence, the concept of coupling of multiple small GTPases might be more universal than currently anticipated. While these studies further provide evidence for the intriguing possibility that endosomes serve as a hub for Rho GTPase activation and spatiotemporal signal generation, they also raise the question about how this mechanism is controlled. The kinase LRRK2 is unusual in that it harbors a GTPase (ROC) domain. LRRK2 was found to localize to endosomes and to play an important role in negatively controlling Rac1 activity on this site (Schreij et al., 2015). Loss of LRRK2 resulted in hyperactive Rac1 and loss of dendritic spines in neuronal dendrites. It is unclear whether LRRK2 regulates Rac1 through phosphorylation, or through a scaffolding effect. There is evidence for phosphorylation-dependent regulation of Rho GTPases (Kwon et al., 2000; Schoentaube et al., 2009; Schwarz et al., 2012). LRRK2 also possesses GTPase activity (Liu and West, 2017) and it remains to be tested whether this activity is involved in Rac1 regulation. Notably, LRRK2 is mutated in familial Parkinson’s disease and was very recently shown to be activated by the Rab29 on endosomes as well as the *trans-*Golgi network (Purlyte et al., 2018). This provides new avenues for future investigations of crosstalk of Rac1 or Cdc42 with Rab29 and linking this to the pathogenesis of Parkinson’s disease.

Rac1 and Cdc42 signaling from endosomes extends beyond Rab5-positive endosomal entities. For instance, Rab11-positive recycling endosomes were shown to traffic Rac1 to immunological synapses (Bouchet et al., 2016). The interaction of Rac1 with Rab11 on recycling endosomes was facilitated by a Rab11 effector FIP3. This Rab11-FIP3-Rac1 tripartite complex controlled T-cell spreading, cortical rigidity and immunological synapse symmetry in a manner dependent on Rac1 (Bouchet et al., 2016). The effects of FIP3 on T-cell spreading and synapse symmetry were Rac1 dependent, because this effect was abolished in the presence of Rac1 inhibitor, NSC23766. We note here that this inhibitor disrupts the interaction of Rac1 with its GEF Tiam/Trio. Thus, it is possible that FIP3 recruits Tiam/Trio to endosomes to mediate localized Rac1 activation. Alternatively, it is possible that Rac1 is recruited to recycling endosomes in its active form. In some cases, endocytosis of certain cell surface receptors may initiate endosomal Rac1 activation. Indeed, c-Met internalization and its trafficking to perinuclear endosomes in response to HGF-stimulation was essential for Rac1 activation (Menard et al., 2014). Notably, optimal Rac1 activation required interaction of c-Met with Rac1 specific GEF Vav2 in the perinuclear endosomes to initiate Rac1-driven cell migration (Menard et al., 2014). Late endosomal Rac1 together with Cdc42 also responds to growth factor independent signals. Using fluorescence resonance energy transfer (FRET)-based biosensors, it was shown that constitutively active Rac1 and Cdc42 promoted actin-nucleation events to drive sorting of cargo into intraluminal vesicles (Kajimoto et al., 2018). Interestingly, this process was initiated by sphingosine 1-phosphate signaling to multivesicular endosomes localized Rac1 and Cdc42, and also required activity from GEFs PLEKHG2 and P-Rex1 (Kajimoto et al., 2018).

Finally, the Rho GTPase family members TCL and TC10 have both been described to reside in early endosomal compartments to control endocytic pathway (de Toledo et al., 2003; Kawase et al., 2006). Apart from that, TCL and TC10 have been relatively underexplored and a clear picture of signaling originating from endosomal pools of these two Rho GTPases is lacking. The activity of TC10 on exocytic vesicles was reported using a FRET-based probe. It was shown that TC10 activity drops sharply just before fusion of the vesicle with the plasma membrane (Kawase et al., 2006). This result indicates that GTP hydrolysis by TC10 is a critical step for vesicle exocytosis. This role of TC10 appears to be applicable to several types of vesicles such as delivery of EGFR to the cell surface (Kawase et al., 2006) as well as translocation of the glucose transporter GLUT4 to the cell surface upon insulin stimulation (Chiang et al., 2001). Because TC10 was also shown to localize to subdomains of the plasma membrane (Liu and West, 2017) it remains to be determined which pool of TC10 regulates exocytic vesicle trafficking.

Taken together, these studies establish that Rho GTPases and their GEFs/GAPs assemble into signaling entities at early/late endosomes (Figure 1). However, it is unclear whether active Rho GTPases at the early/late endosomal compartments stem from plasma membrane internalized active pool. So far, the requirement of GEFs for optimal endosomal Rho GTPase signaling output points toward active recruitment of Rho GTPases to the endosomes. However, such a demonstration by selective manipulation of local pools of GEFs in contrast to depletion of total cellular pools of GEFs is currently missing.

## Rho GTPase Signaling From the Golgi

**FIGURE 2:**
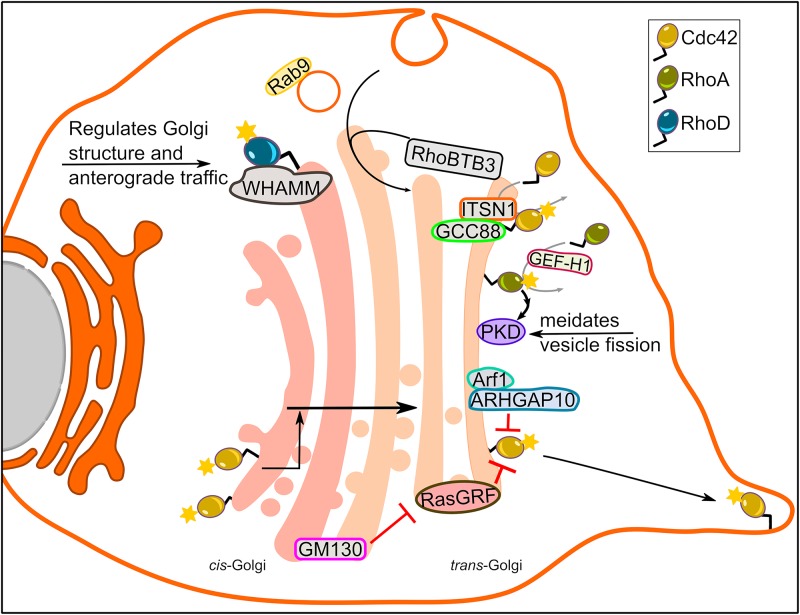
Rho GTPase signaling from the Golgi complex. A number of Rho family members localize to the Golgi apparatus and regulate Golgi morphology, intra-Golgi trafficking and actin dynamics at the Golgi. The figure highlights function of some of the Rho GTPases at the Golgi.

Over two decades ago, Cdc42 was already noticed to localize to the Golgi (Erickson et al., 1996). Since then a number of other Rho GTPase family members, their GEFs and GAPs, and interaction partners of Rho GTPases have been detected at the Golgi apparatus (Figure 2). At the Golgi, Cdc42 interacts with components of the COPI coat, which was originally shown to be important for cellular transformation (Wu et al., 2000). A major function of coatomer is to mediate formation of COPI vesicles for retrograde transport from the Golgi back to the ER (Letourneur et al., 1994). Accordingly, Cdc42 was shown to regulate membrane deformation by coatomer components (Park et al., 2015). However, rather than affecting COPI transport back to the ER, Cdc42 appeared to promote intra-Golgi anterograde transport. Thus, active Cdc42 might introduce a bias toward anterograde versus retrograde COPI trafficking (Park et al., 2015). It should be mentioned that these results were primarily obtained with a fast cycling mutant of Cdc42 (Cdc42-F28L) and that it remains to be shown whether Cdc42 can introduce this traffic bias under physiologic conditions. Notably, the fact that several tumors exhibit secretion of ER chaperones, might be interpreted as a failure of COPI-based retention. Future work should test the possibility whether higher Cdc42 activity at the Golgi is involved in this phenomenon.

An important question is whether Cdc42 is active at the Golgi, which is best answered by live cell imaging. The first detection of active Cdc42 at the Golgi was made possible by FRET-based reporters (Nalbant et al., 2004). However, it remained unclear whether this pool is functionally relevant for canonical Cdc42-related processes such as regulation of the cytoskeleton, cell polarity or cell migration. An early indication that this pool is functionally relevant for these processes was the finding that coatomer-bound (i.e., Golgi localized) Cdc42 stimulates actin assembly, but inhibits recruitment of the molecular motor dynein to the nascent vesicles (Chen et al., 2005). This observation suggests a model where Cdc42 disassociates from the coatomer once the vesicle formation is completed, allowing dynein to bind and transport vesicles. In addition, the Golgi pool of Cdc42 was reported to mediate dynein and microtubule-dependent Golgi positioning in directionally migrating cell (Hehnly et al., 2010). Later, the Golgi matrix protein GM130 was proposed to act as a factor that selectively regulates the Golgi pool of Cdc42 without affecting the plasma membrane (Baschieri et al., 2014). GM130 binds to RasGRF, which was previously shown to inhibit Cdc42 activity (Calvo et al., 2011). Using GM130 to specifically modulate Cdc42 activity at the Golgi, it was demonstrated that this pool acts as a reservoir that supplies the leading edge plasma membrane of directionally migrating cells with active Cdc42 (Baschieri et al., 2014). This was in agreement with previous reports showing that cytoplasmic vesicles carrying Cdc42 are delivered to the leading edge (Osmani et al., 2010).

Another interesting question is whether the Golgi harbors GAPs and GEFs that would mediate Cdc42 activity at this organelle. A GAP for Cdc42, ARHGAP10, was reported to be recruited to the Golgi by active Arf1 (Dubois et al., 2005). It was revealed that active Arf1 via its interaction with ARHGAP10 controls Cdc42 activity at the Golgi, and in this way regulates Golgi structure and actin cytoskeleton dynamics at the Golgi (Dubois et al., 2005). Earlier report suggested that the GEF Tuba might play a role in modulating Cdc42 activity at the Golgi (Kodani et al., 2009). However, four independent groups have failed to localize Tuba to the Golgi, but rather found it to be confined to cytoplasmic vesicles or the cell surface (Salazar et al., 2003; Kovacs et al., 2006; Baschieri et al., 2014; Bruurs et al., 2018). Very recently, the Golgi matrix protein GCC88 was found to interact with the long form of intersectin-1, a Cdc42 GEF (Makhoul et al., 2019). Intersectin-1 was convincingly localized to the Golgi providing us with an excellent candidate that might mediate local activation of Cdc42. Further supporting a role for intersectin-1 at the Golgi is an earlier report showing that a small molecule (ZCL278) that inhibited Cdc42-intersectin1 interaction disrupted Golgi structure (Friesland et al., 2013). It will be interesting to see whether this drug could be used to test some of the aforementioned effects of Golgi-based Cdc42 functions such as COPI transport or cell transformation.

In addition to Cdc42, active RhoA have also been localized to Golgi (Quassollo et al., 2015). Using FRET-based biosensors, active RhoA was detected at Golgi outposts (GOPs) in neuronal dendrites (Quassollo et al., 2015). Reportedly, the biogenesis of GOPs occurs from dendrite-localized ER exit sites (Horton et al., 2005). However, it remained unclear whether GOPs could emanate through fission from the somatic Golgi. RhoA at the Golgi was activated downstream of lysophosphatidic acid initiating the activation of a cascade involving ROCK, LIMK1 and PKD1, thereby resulting in tubulation of the somatic Golgi and elongation of these tubules into dendrites (Quassollo et al., 2015). RhoA was also involved in activating dynamin-mediated fission events in dendrites leading to separation of the Golgi tubules and the formation of GOPs. Whether this is a main pathway for GOP formation has to be tested in the future. Because GOPs are important for dendrite formation and branching, it is conceivable that they play an important role in synaptic integration and plasticity. Thus, potential roles of Golgi-based signaling of RhoA in these processes merits future investigations.

A very recent example of an active role of RhoA at the Golgi is the identification of a cascade triggered by protease activated receptors at the cell surface that activate a RhoA GEF called GEF-H1. GEF-H1 mediates RhoA activation at the *trans-*Golgi, which in turn activates PKD, a Ser/Thr kinase of great importance for the biogenesis of post-Golgi carriers. Thereby, RhoA was shown to regulate cargo delivery for localized exocytosis at focal adhesions (Eisler et al., 2018).

More recently, Golgi localization of RhoD and its role in maintaining Golgi homeostasis was reported (Blom et al., 2015). Endogenous RhoD colocalized with the Golgi resident proteins, whereas ectopic expression of constitutively active and inactive RhoD alone or with its binding partner WHAMM (WASP homolog associated with actin, membranes, and microtubules) led to Golgi fragmentation (Blom et al., 2015). Using temperature sensitive VSV-G to monitor trafficking, it was found that RhoD deregulates anterograde vesicular transport from the endoplasmic reticulum (ER) to the plasma membrane. In cells where RhoD activity was perturbed, VSV-G was scattered in vesicular structures positive for GM130 that were sensitive to endoglycosidase-H cleavage, indicating that the VSV-G containing Golgi derived vesicles were not fully functional (Blom et al., 2015). This suggests a role for RhoD in the secretory pathway.

The recently identified RhoBTB-1,-2, and -3 are much larger than classical small GTPases, possess additional domains and are not regulated by the conventional GTPase cycle (Aspenstrom et al., 2007; Ji and Rivero, 2016). In fact, RhoBTB3 – the only RhoBTB family member that was reported to localize to Golgi – functions as an ATPase (Espinosa et al., 2009). RhoBTB3 is anchored at the *trans-*side of the Golgi, where it functions as an effector for Rab9, a GTPase localized on late endosomes that traffics to the *trans-*Golgi. Arrival of these Rab9-positive carriers to the Golgi, induces activation of the ATPase activity of RhoBTB3, which is required to remove the coating off these vesicles, thereby preparing them to fuse with the Golgi (Espinosa et al., 2009). Corroborating this, it was further demonstrated that Golgi-residing RhoBTB3 is important for maintaining Golgi architecture since its depletion resulted in Golgi fragmentation (Lu and Pfeffer, 2013). At the Golgi RhoBTB3 is part of a Cul3-RING-E3 ubiquitin ligase complex, which binds Cyclin E and targets its proteasomal degradation (Lu and Pfeffer, 2013). This finding highlights that a Golgi-localized signaling molecule plays a role in the cell cycle by regulating G1-S-phase entry, a phase where Golgi has not been involved previously.

A major function of Rho GTPases is to regulate the cytoskeleton as well as molecular motors. The actin-nucleating Arp2/3 complex was shown to localize to the Golgi and to play a role in its polarization during cell migration (Magdalena et al., 2003). The dynamics of Arp2/3 at the Golgi were later shown to be regulated by the Golgi-pool of Cdc42 (Dubois et al., 2005). Cdc42 was also shown to regulate actin-assembling formin family members FMNL -2 and -3 at the Golgi and to thereby regulate the architecture of this organelle (Kage et al., 2017). Further evidence linking Cdc42 and Golgi architecture via regulation of actin dynamics was provided by identifying the Cdc42 exchange factor ITSN-1 as an interaction partner for the Golgi matrix protein GCC88 (Makhoul et al., 2019). Importantly, this interaction was suggested to play a role in Golgi dispersal that is observed in neurodegeneration.

## Rho GTPase Signaling From Mitochondria

The first ever description of mitochondria localized Rho GTPase was provided by Boivin and Beliveau (1995). The authors subjected outer cortical region of kidney from Sprague–Dawley male rats to subcellular fractionation and probed for RhoA, Cdc42 and Rac1 in different subcellular fractions. While all three Rho GTPases were detected in plasma membrane and cytosolic fractions, only Rac1 was detected in mitochondria-enriched fractions (Boivin and Beliveau, 1995). Corroborating this, Velaithan et al. (2011) showed direct physical interaction between mitochondrial Rac1 and Bcl-2 in human cancer cell lines and clinical biopsies from B-cell lymphoma patients. A year later, Rac1 was also detected in mitochondria in alveolar macrophages isolated from asbestosis patients (Osborn-Heaford et al., 2012). This study further revealed that the C-terminal cysteine (Cys-189) residue of Rac1 is required for its mitochondrial import. Subsequent studies have since included neuronal cells in the repertoire of cell types exhibiting mitochondrial Rac1 (Natsvlishvili et al., 2015; Pan et al., 2018).

**FIGURE 3:**
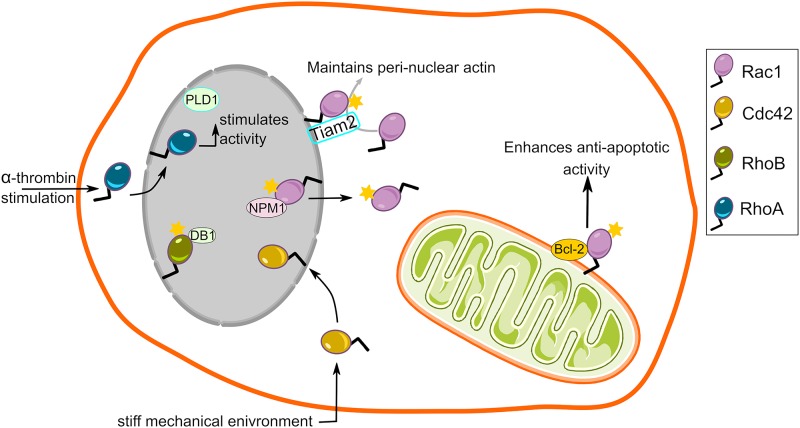
Rho GTPase signaling from the mitochondria and the nucleus. Rac1, a member of the Rho family, localizes to the mitochondria and interacts with BCL-2 to enhance its anti-apoptotic activity. It is unknown whether other members of Rho family localize to the mitochondria. Nuclear localization and a wide range of functional roles has also been described for several members of the Rho GTPase family.

In addition to describing localization of Rac1, these studies also provide compelling evidence for Rac1 signaling on mitochondria. The direct interplay of Rac1 and Bcl-2 at mitochondria maintained a mild pro-oxidant intracellular milieu through increased intracellular superoxide levels, successively promoting the death inhibitory activity of Bcl-2 (Velaithan et al., 2011; Figure 3). Interestingly, inhibition of Rac1-Bcl-2 interaction in lymphoma cells restored death signaling in response to common chemotherapeutic agents (Velaithan et al., 2011). Elevated mitochondrial Rac1 activity in alveolar macrophages via its interaction with cytochrome c, increased oxidative stress and contributed to the development of pulmonary fibrosis (Osborn-Heaford et al., 2012). Furthermore, increased Rac1 signaling was proposed to play important roles in the regulation of neuroplasticity and prevention of apoptosis and autophagy via its association with sigma-1 receptor, inositol 1,4,5-trisphosphate receptor and Bcl-2 at the mitochondrial membrane (Natsvlishvili et al., 2015). In contrast to these findings, interference of Rac1-Bcl-2 complex either by inhibition or siRNA mediated depletion of Rac1 relieved mitochondrial oxidative stress and promoted neuronal survival in a focal cerebral ischemia *in vivo* in a diabetic rat model and a hyperglycemia-exposed PC-12 cell *in vitro* model (Pan et al., 2018). Hence, Rac1 signaling at the mitochondria could produce cell-type specific outcomes under different conditions.

Generally, mitochondria have largely been overlooked as sites for Rho GTPase signaling. Whether members of Rho GTPase family other than Rac1 also localize to mitochondria and might have been missed because of their rapid shuttling in and out of the mitochondria remains a subject for future studies.

## Rho GTPase Signaling From Nucleus

Because the outer nuclear membrane is part of the ER and it also is a membrane enclosed organelle, we will briefly review evidence for Rho GTPase signaling in and from this location. The C-terminal polybasic region of Rac1 harbors functional nuclear localization signals (K-K-R-K and K-R-K-R) that promote its nuclear translocation (Lanning et al., 2004; Navarro-Lerida et al., 2015). Reportedly, Rac1 also contains two internal nuclear export motifs (Navarro-Lerida et al., 2015). It was revealed that interaction of Rac1 with nucleophosmin-1 mediates its efficient nuclear export (Navarro-Lerida et al., 2015; Figure 3). Consequently, wealth of data describe nucleocytoplasmic shuttling of Rac1 and postulate functional implications of nuclear Rac1 signaling (Kraynov et al., 2000; Michaelson et al., 2008; Menard et al., 2014; Navarro-Lerida et al., 2015; Woroniuk et al., 2018). Such a nuclear localization signal is also present in the C-terminal polybasic region of several other Rho GTPase family members (Williams, 2003). Whether other Rho GTPases also shuttle in and out of the nucleus in a manner similar to Rac1 remains to be investigated. Rac1 cycling in and out of the nucleus seemingly depends on the cell cycle, with increased nuclear Rac1 during the late G2 phase (Michaelson et al., 2008). In addition to Rac1, its GEF Tiam2 is also present at the outer nuclear membrane (Woroniuk et al., 2018), providing the basis for Rac1 signaling at the nuclear envelope (Figure 3). The question next arises is what functional consequences does Rac1 signaling in nucleus or on its envelope might have. Given its pivotal role in actin dynamics, Rac1 signaling in the nucleus could lead to changes in nuclear shape and position in actin-dependent fashion. This could have consequences for cancer cell invasion since structural changes in shape and size, and deformity of the nucleus are decisive factors for invasion through tight gaps in the extracellular matrix (Friedl et al., 2011). An additional effect of Rac1 signaling was altered nuclear membrane fluidity and order (Navarro-Lerida et al., 2015), which might further be an important factor regulating the ability of the nucleus to deform during invasion. The actin mesh around the nucleus might also play a role in positioning of this organelle, because depletion of Rac1 GEF Tiam2 resulted in a failure to position the nuclei with cellular axis of migration (Woroniuk et al., 2018). Moreover, aggressive tumors display higher nuclear Rac1, a phenomenon that results in increased invasiveness *in vitro* by potentiating cytoplasmic RhoA signaling (Navarro-Lerida et al., 2015). Alternatively, Rac1 may directly interact with nuclear proteins to induce actin-independent changes in cells. On one hand, active Rac1 directly interacts with STAT3 and regulates its activity by promoting its phosphorylation (Simon et al., 2000). On the other hand, Rac1 interaction with nucleophosmin-1 attenuates Rac1 signaling and inhibits cell spreading (Zoughlami et al., 2013). Moreover, increased Rac1 shuttling into the nucleus accelerates cell division (Michaelson et al., 2008). Finally, a fraction of active monomeric Rac1 segregated in the nucleus from dimeric and inactive Rac1 in the cytoplasm upon induction of DNA damage (Hinde et al., 2014).

Despite lacking a canonical nuclear localization signal, RhoA has been reported to translocate to the nucleus (Baldassare et al., 1997; Dubash et al., 2011; Figure 3). When quiescent fibroblasts were stimulated with α-thrombin, RhoA was found to translocate to the nucleus and stimulate enzymatic activity of PLD leading to the production of phosphatidic acid and diacylglycerol (Baldassare et al., 1997). The precise biological consequences of these lipid species in the nucleus remains to be fully understood. Later, Garcia-Mata and co-workers also demonstrated that a pool of RhoA is present in the nucleus with a subset of its GEFs (Net1, Ect2) and GAPs (DLC1, p190 RhoGAP). Notably, RhoA and Net1 activity selectively increased in the nucleus upon DNA damage implicating nuclear Net1/RhoA activity in DNA damage signaling (Dubash et al., 2011).

Another Rho GTPase with potential nuclear effects, but no clear nuclear localization signal is RhoB, which was shown to localize to the nuclear envelope, and to interact with and regulate the transcription factor DB1 (Lebowitz and Prendergast, 1998; Gerald et al., 2013; Figure 3). The RhoB-DB1 interaction was important for sprouting and proliferation in primary human blood endothelial cells (angiogenesis) in favor of lymphatic endothelial cells (lymphangiogenesis). Accordingly, RhoB knockout mice exhibited reduced angiogenesis but enhanced lymphangiogenesis in response to wounding (Gerald et al., 2013). Neither the stimulus that triggers RhoB translocation, nor the temporal dynamics of this subcellular pool were determined. Elucidating these details will be important to gain a full understanding of spatio-temporal RhoB signaling.

A recent study has described a biologic role for nuclear Cdc42 (Liu et al., 2018). When cultured under stiff mechanical environment, Cdc42 translocated from cytoplasm to the nucleus in tumor repopulating cells (i.e., cancer cells with stem-like properties) (Liu et al., 2018). The nuclear translocation of Cdc42 elevated expression of Tet2, an epigenetic modifier involved in chromatin methylation. Tet2 expression leads to increased expression of p21 and p27, which induce a G1-phase arrest and thus dormancy. The fact that stiffness-mediated dormancy was observed *in vivo* suggests that nuclear Cdc42 activity might also be relevant in cancer (Liu et al., 2018). Future work is needed to test how essential Cdc42 is for this process and whether identifying drugs that inhibit nuclear translocation of Cdc42 might be useful for cancer therapeutics.

## Concluding Remarks

It is becoming increasingly apparent that signals emanating from distinct subcellular pools of Rho GTPases is spatially and temporally regulated to generate diverse physiological outcomes. Owing to the recent advancements in Rho GTPase biosensors and microscopy techniques, our knowledge of the complexity of Rho GTPase signaling in time and space has significantly advanced. Nevertheless, many new and interesting questions are yet to be addressed. For example, our understanding of any potential Rho GTPase signaling originating at the ER, the largest organelle in the cell, remains primitive. ER extends throughout the cytoplasm and forms membrane contact sites with endosomes, Golgi, mitochondria and plasma membrane. Membrane contact sites are known to play well-defined roles in bidirectional transport of molecules as well as signal transmission. Since Rho GTPases are able to signal from endosomes, Golgi, mitochondria and plasma membrane, it is tempting to speculate that they may participate in the biogenesis of membrane contact sites, or use them as passages to change subcellular localization. Such speculations are supported by the observation in yeast that the Rho-like GTPase Gem1 (the homolog of mammalian Miro1) is localized to the ER-mitochondrial contact sites (Kornmann et al., 2011). The molecular machinery that recruits Rho GTPases and their GEFs/GAPs to distinct endomembranes has yet to be thoroughly characterized. Finally, we need to incorporate the diverse Rho GTPase signaling units together to better understand their crosstalk and biological outcome as a whole. This makes it clear that using drugs or tools that globally activate or inhibit Rho GTPases is unlikely to be of great use. Future efforts should focus on identifying tools and methods to specifically modulate subcellular pools.

## Author Contributions

SP and HF researched the relevant literature, conceived, and prepared the manuscript.

## Conflict of Interest Statement

The authors declare that the research was conducted in the absence of any commercial or financial relationships that could be construed as a potential conflict of interest.
